# Associations between dietary antioxidant vitamins and risk of glioma: an updated systematic review and meta-analysis of observational studies

**DOI:** 10.3389/fnut.2024.1428528

**Published:** 2024-08-06

**Authors:** Ya-Jun Ni, Yi-Qian Huang, Lin Yu, Xiao-Yan Zhang, Qin Zhu, Long Shu, Lun Zhang

**Affiliations:** ^1^Department of Anesthesia Operation, Zhejiang Hospital, Zhejiang Hospital, Hangzhou, Zhejiang, China; ^2^Department of Nutrition, Zhejiang Hospital, Hangzhou, Zhejiang, China; ^3^Department of Digestion, Zhejiang Hospital, Hangzhou, Zhejiang, China

**Keywords:** antioxidant vitamins intake, glioma, meta-analysis, systematic review, epidemiology

## Abstract

**Background:**

Epidemiological studies investigating the potential associations between antioxidant vitamins intake and risk of glioma have yielded inconsistent results. To address this, we carried out a systematic review and updated meta-analysis to explore the relationship between dietary antioxidant vitamins intake and risk of glioma.

**Methods:**

We comprehensively searched electronic databases including PubMed, Web of Science, Embase, Scopus, China National Knowledge Infrastructure (CNKI) and Wan fang Data from their inception to March 2024. We employed fixed-effects or random-effects models to estimate the pooled relative risks (RRs) and 95% confidence intervals (CIs) for the associations between dietary antioxidant vitamins intake and risk of glioma. Publication bias was assessed through the visual inspection of the funnel plots and quantified by the Begg's and Egger's tests. Heterogeneity across studies was assessed using the Cochran's Q test and I-square (I^2^). Additionally, subgroup and sensitivity analyses were performed to explore potential sources of heterogeneity and evaluate the robustness of the results.

**Results:**

Overall, a total of 15 articles involving 3,608 glioma cases and 771,930 participants were included in the final analysis. The pooled analyses revealed that the highest intake of vitamin C significantly reduced the risk of glioma (RR = 0.78; 95%CI: 0.63–0.96; *P* = 0.022), compared to the lowest intake. However, no significant associations were observed between vitamin A and vitamin E intake and the risk of glioma (*P*>0.05). Subgroup analyses revealed the inverse association between vitamin C intake and risk of glioma in the population-based case-control studies (RR = 0.82; 95%CI: 0.68–1.00, *P* = 0.049) and study quality <7(RR = 0.52, 95%CI: 0.29–0.92, *P* = 0.025).

**Conclusion:**

Our findings show that higher intake of vitamin C is strongly associated with a reduced risk of glioma, although a dose-response relationship was not evident. Future large-scale prospective studies are warranted to confirm these findings.

## Introduction

Glioma is a central nervous system (CNS) tumor that originates from the glial cells of human brain, accounting for approximately 80% of adult malignant brain cancers (1). According to the reports by the International Agency for Research on Cancer (IARC) in 2020, the global incidence of brain cancer is 3.9 per 100,000 for males and 3.0 per 100,000 for females (2). In Iran, the latest data estimated that the incidence of brain tumors was 2.92 and 2.46 per 100,000 in men and women, respectively (3). In contrast, the annual incidence of glioma in the Chinese population is about 7.01 per 100,000, significantly higher than in Iran (4). Despite extremely low incidence of glioma, the main concerns are its high mortality rate and poor prognosis (5). It has been reported that more than 97% of glioma patients die within 5 years of diagnosis (6). Also, the standard clinical treatment, including surgical resection combined chemoradiotherapy for glioma is not satisfactory and it has incurred a severe disease burden (7).Thus, early diagnosis and prevention of glioma have become the important strategies for fighting against this disease.

Over the past few decades, there has been a lack of comprehensive understanding of the etiology and treatment of glioma. At present, only exposure to high-dose ionizing radiation has been identified as the clearly established environmental risk factors for this type of cancer (8). Of concern, dietary antioxidants have long been recognized as important protective factors for various types of cancer, including breast cancer (9), colocteral cancer (10) and liver cancer (11). Nevertheless, less research attention has been given to the impact of dietary antioxidant vitamins on brain tumors, particularly glioma. A recently published systematic review and meta-analysis of 24 observational studies on healthy dietary patterns, foods and risk of glioma showed that antioxidant-rich foods, such as vegetables and fruits, were significantly associated with the lower risk of glioma (12). In addition to this, limited epidemiological studies have thus far been published to assess the relationship between dietary antioxidant vitamins intake and risk of glioma (13–27). But, these epidemiological studies have produced inconclusive results. Although some previous studies have revealed the significant protective role of high antioxidant vitamins intake against glioma (17, 18, 27), other studies reported the apparent positive or null findings (13–16). For example, a recent hospital-based case-control study by Heydari et al., concluded that dietary intake of vitamin C was inversely associated with risk of glioma in Iranian adults (OR = 0.14; 95%CI:0.05–0.36) (18). Similarly, in the San Francisco Bay Area Adult Glioma Study, Tedeschi-Blok et al. also found that higher intake of vitamin A and vitamin C were associated with a reduced risk of glioma (19). By contrast, in the prospective NIH-AARP Diet and Health Study, Dubrow and colleagues failed to find any significant effect of vitamin C on glioma risk (16). Still, an earlier meta-analysis of 13 articles published in 2015, concluded that the highest vitamin C intake was significantly associated with a decreased risk of glioma (RR = 0.86; 95%CI: 0.75–0.99) (28). However, in Zhou's meta-analysis, the outcome of interests were glioma, meningiomas and other brain tumors, and two of included studies reported the effects of vitamin C on brain tumors in children. Since the publication of that meta-analysis, several new epidemiological research on the relationship between antioxidant vitamins intake and glioma risk have been published (18, 25–27). To date, there is no comprehensive and updated meta-analysis evaluating the relationship betwen vitamin C intake and glioma risk. Furthermore, no firm judements thus far have been made regarding the associations between other antioxidant vitamins, including vitamin A and vitamin E intake and risk of glioma. In view of the rarity of glioma, the majority of available studies on the associations between dietary antioxidant vitamins intake and glioma included a relatively small number of cases, which limited the statistical power of any single study to explore the exact association. Therefore, to provide a more comprehensive understanding of the effect of dietary antioxidant vitamins on the risk of glioma, we conducted an updated systematic review and meta-analysis to synthesize the available literature published up to March, 2024.

## Materials and methods

The current systematic review and meta-analysis was carried out in accordance with the Preferred Reporting Items for Systematic Reviews and Meta-Analyses (PRISMA) guidelines (29). However, the protocol for this review has not yet been registered in the International Prospective Register of Systematic reviews (PROSPERO).

### Search strategy

A thorough literature search was performed in PubMed, Web of Science, Embase, Scopus, China National Knowledge Infrastructure (CNKI) and Wanfang Data from inception to March 2024, on human studies providing data for the highest compared with the lowest category of antioxidant vitamins intake. The following search terms were used: (“diet” OR “nutrition” OR “nutrient” OR “vitamin C” OR “vitamin E” OR “vitamin A” OR “antioxidants”) AND (“glioma” OR “glioblastoma” OR “brain cancer” OR “brain tumor”). The complete search strategy is shown in the Supplementary Table S1. During the search process, only articles in the English or Chinese were considered. Additionally, we identified potentially eligible articles by manually searching the reference lists of all retrieved articles and reviews. Any discrepancies in selecting articles were resolved by consensus or by consulting a third investigator (L.S.).

### Studies included criteria

Two independent reviewers (Y-.J N and L.Z.) reviewed the titles and abstracts of articles retrieved in an initial search to identify eligible studies that reported antioxidant vitamins intake and glioma risk. Differences between the two independent reviewers were resolved by consensus and together with a third reviewer if necessary. All reviewers agreed on the relevant articles, and full-text versions of articles were reviewed against inclusion and exclusion criteria for this meta-analysis. Studies that fulfilled the following criteria were included in this meta-analysis: (1) the study design was observational study, e.g., case-control, nested case-control or cohort; (2) the article was published in the English or Chinese languages; (3) the exposure of interest was dietary antioxidant vitamins, including vitamin A, C and E; (4) the outcome of interest in this study was glioma; (5) multivariate-adjusted relative risk (RR) or HR or OR with their corresponding 95% confidence intervals (CIs) were provided (or sufficient data to calculate them); (6) If the data in original article lacked sufficient detail, the corresponding author of the included studies was contacted for additional information by email. Studies were excluded if they met one of based on the following criteria: (1) written in a language other than English or Chinese; (2) not performed on humans; (3) non-observational study, e.g. reviews, case reports, letters and clinical trials; (4) studies with insufficient data for the highest category vs. lowest intake of vitamin A, vitamin C or vitamin E. The PICOS criteria for inclusion and exclusion of studies is shown in Supplementary Table S2.

### Data extraction

Two researchers (Y.-Q.H and X.-Y.Z) independently conducted the data extraction using a standardized form. For each selected studies, the following information were extracted: first author's name, publication data, country, study design, age, sex, number of cases and controls or participants, antioxidant vitamins source, confounders adjusted for, and effect sizes and 95% CI for the highest category vs. lowest intake. In the case of presenting gender-stratified effect sizes, we treated them as two separate studies.Any disagreements in data extraction were resolved by discussion for reaching a consensus.

### Quality assessment

The Newcastle-Ottawa Quality Scale (NOS) was used by two reviewers (L.Y. and Q.Z.) to separately evaluate the quality of the included studies in the present meta-analysis (30). According to this scale, each study could be assigned a maximum score of 9 points for three main domains: selection (range 0–4 points), comparability (range 0–2 points), and assessment of outcomes (range 0–3 points). Studies with the score of 7–9 points, 4–6 points, and 0–3 points, were regarded as being high, medium, and low quality, respectively (31). Disagreements were resolved through discussion to reach a consensus.

### Statistical analysis

Because the prevalence of glioma is relatively low, ORs and HRs are considered as approximations of RRs, and were combined with RRs (32). The Cochran's Q test and I^2^ statistics were used to evaluate the inter-study heterogeneity. In the current meta-analysis, a random-effects model (DerSimonnian and Laird method) was used to pool RRs when significant heterogeneity was detected, as measured by an *I*^2^> 50% or *P*-values of Cochran's Q test <0.10. Otherwise, the fixed-effects model was adopted (33). If the results showed significant heterogeneity (*I*^2^>50%), the potential sources of between-study heterogeneity were examined by using prespecified subgroup analyses. Moreover, the analysis of sensitivity analysis was also performed to assess the stability of the pooled results by omitting one study at a time. Potential publication bias was assessed through the visual inspection of the funnel plots and quantified by the Begg's and Egger's tests, respectively (34). If there was evidence of publication bias, we would apply the trim and fill method to adjust the asymmetry of the funnel plot by imputing missing study data (35).The statistical analyses were carried out using the STATA software, version 12.0 (Stata Corp, College Station, TX, USA). All reported *P*-values were two-sided, and the significance level was set at <0.05 for statistical analyses.

## Results

### Overview of included studies for this systematic review and meta-analysis

**Figure 1:**
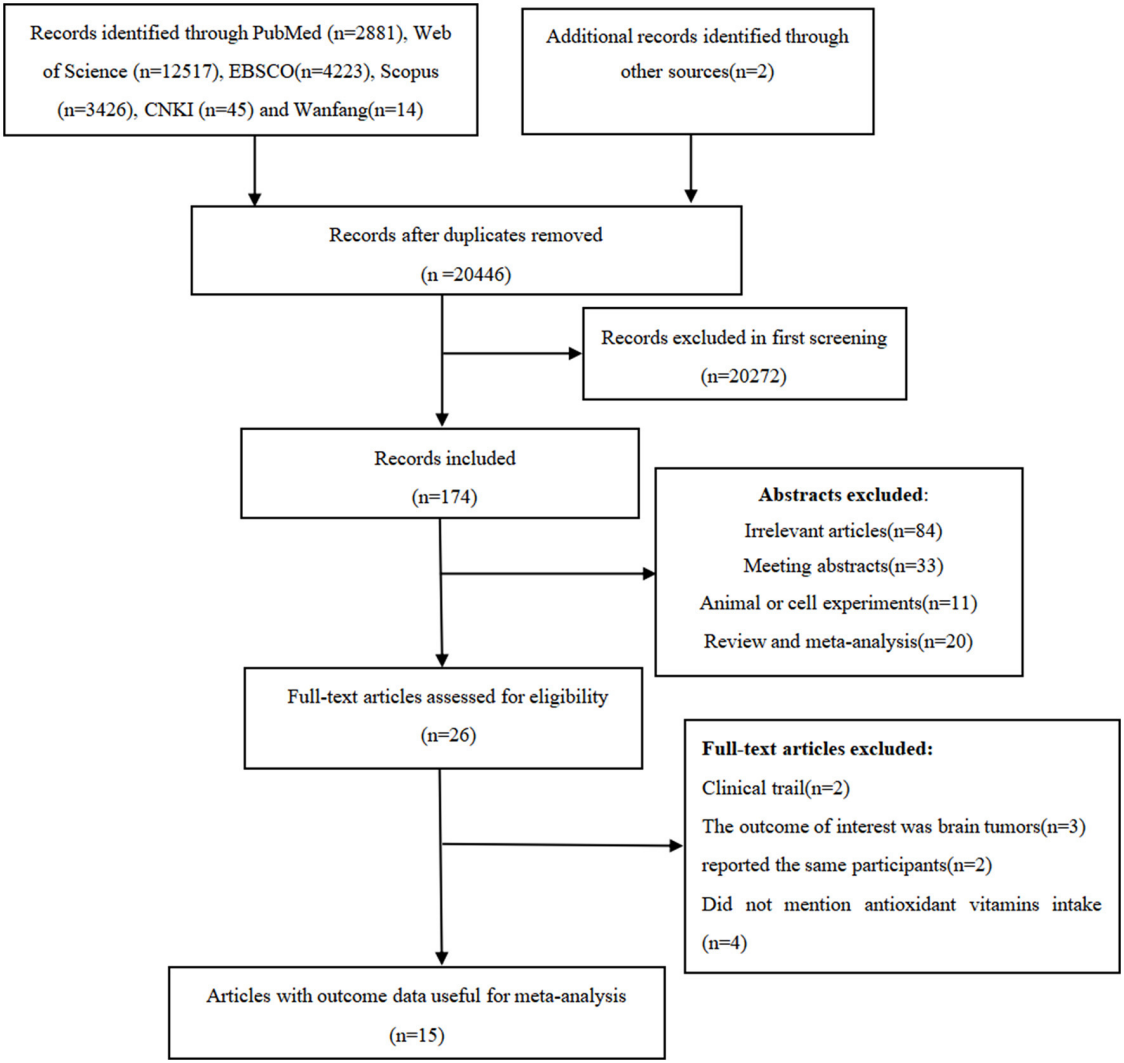
The detailed process of literature search and selection.

We identified 2,881 articles from PubMed, 12,517 articles from Web of Science, 4,223 articles from EBSCO, 3,426 articles from Scopus, 45 articles from CKNI, and 14 articles from Wan fang database. After removal of the duplicate records, 20,446 articles remained. Whereafter, screening the titles and abstracts of the remmaining articles led to the exclusion of 20,272 articles because they did not examine the associations between antioxidant vitamins and glioma risk, and the full-texts of the remaining 26 articles were carefully checked for eligibility. Out of 26 articles considered for full-text assessment, 11 articles were excluded for the following reasons: clinical trials (*n* = 2), the outcome of interest was brain tumors (*n* = 3); did not mention antioxidant vitamins intake (*n* = 4); and reported the same participants (*n* = 2). Ultimately, 15 articles, including two prospective cohort (16, 21) and 13 case-control studies (13–15, 17–20, 22–27), were considered for the final analysis. The detailed process of literature search and selection can be seen in Figure 1.

### Study characteristics

The baseline characteristics from all included studies evaluating the associations of dietary antioxidant vitamins and glioma risk are shown in Table 1. Overall, 15 articles with 772,616 participants and 3,951 glioma cases were included in this systematic review and meta-analysis. The majority of the included studies were case-control in design (13–15, 17–20, 22–27), and two of them were cohort design (16, 21). All these studies were published in English or Chinese between 1993 and 2024. The age of participants ranged from ages 18 to above. Of the 15 included studies, nine were conducted in the United States (16, 17, 19–25), two in Iran (18, 26), two in China (15, 27), one in Germany (13), and one in Australia (14). Overall, according to NOS, 13 articles were classified as of high quality (13, 14, 16–25, 27), and the remaining two articles were of medium quality (15, 26). The NOS scores of the included studies ranged from 7 to 9, with a median value of 8, which suggested the high quality of eligible studies (Table 1).

**Table 1:** Characteristics of included studies on antioxidant vitamins intake and risk of glioma (−2024).

**References**	**Country**	**Study design**	**No. of cases**	**Age range**	**Duration of follow-up**	**Method of dietary assessment**	**Effect size (95%CI)**	**Adjustment or matched for**
Boeing et al. (13)	Germany	Case-control (PCC)	115 cases and 418 controls	25–75 y	-	Interviews	Vitamin C: 0.90 (0.50–1.70)	Age, gender, tobacco-smoking and alcohol consumption
Giles et al. (14)	Australia	Case-control (PCC)	416 cases and 422 controls	20–70 y	-	Questionnaire	Vitamin A:1.28 (0.91–1.79) for males, 1.05 (0.69–1.59) for females; Vitamin C:1.42 (0.86–2.34) for males, 0.62 (0.34–1.13) for females; Vitamin E:1.68 (1.07–2.63) for males, 1.09 (0.62 −1.92) for females	Alcohol and tobacco
Hu et al. (15)	China	Case-control (HCC)	129 cases (73 gliomas, 56 meningiomas), 256 controls	20–74 y	-	FFQ	Vitamin A:0.38 (0.10–1.60) Vitamin C:0.78 (0.2–4.1) Vitamin E:0.16 (0.10–0.50)	Matched to each case by sex, age within five-year intervals and area of residence (same or adjacent city and country).
Dubrow et al. (16)	United States	Cohort	585 cases, 545,770 participants	50–71 y	7.2 y	FFQ	Vitamin C:1.26 (0.96–1.66) Vitamin E:1.17 (0.90–1.53)	Sex, age, race, energy intake, education, height, and history of cancer at baseline
Tedeschi-Blok et al. (17)	United States	Case-control (PCC)	802 cases and 846 controls	≥20 y	-	Questionnaire	Vitamin A:0.65 (0.48–0.88) Vitamin C:0.70 (0.51–0.94) Vitamin E:0.91 (0.62–1.34)	Age, gender, ethnicity, SES, total calories, and supplement use
Heydari et al. (18)	Iran	Case-control (HCC)	128 cases and 256 controls	20–75y	-	FFQ	Vitamin A:0.99 (0.45–2.18) Vitamin C:0.14 (0.05–0.36) Vitamin E:0.83 (0.35–1.97)	Age (continuous), energy intake (kcal/d), sex, BMI, physical activity (continues), family history of cancer (yes/no), family history of glioma (yes/no), marital status (married/single/divorced), education (university graduated/non- university education), high risk occupation (farmer/nonfarmer), high risk residential area (yes/no), duration of cell phone use (continuous), supplement use (yes/no), history of exposure to the radiographic X-ray (yes/no), history of head trauma (yes/no), history of allergy (yes/no), history of hypertension (yes/no), smoking (smoker/nonsmoker), exposure to chemicals (yes/no), drug use (yes/no), personal hair dye use, frequentfried food intake (yes/no), frequent use of barbecue, canned foods and microwave (yes/no)
Blowers et al. (19)	United States	Case-control (PCC)	94 cases and 94 controls	25–74 y	-	Questionnaire	Vitamin A:0.70 (0.30–1.90) Vitamin C:1.50 (0.60–4.10) Vitamin E:2.20 (0.80–5.70)	Matched the patient on age (within 5 years) and race (Black or White).
Chen et al. (20)	United States	Case-control (PCC)	236 cases and 449 controls	≥21 y	-	FFQ	Vitamin A:0.87 (0.41–1.24) Vitamin C:0.90 (0.50–1.50) Vitamin E:0.80 (0.50–1.40)	Age, age squared, gender, total energy intake, respondent type, education level, family history, and farming experience
Michaud et al. (21)	United States	Cohort	335 cases, 219,334 participants	25–75 y	<24 y	FFQ	Vitamin C:0.88 (0.62–1.26); Vitamin E:0.7 (0.67–1.43)	Age and caloric intake (quintiles)
Schwartzbaum et al. (22)	United States	Case-control (HCC)	40 cases and 48 controls	36–69 y	-	FFQ	Vitamin C:0.5 (0.1–4.6)	Age, sex and race.
Lee et al. (23)	United States	Case-control (PCC)	434 cases and 439 controls	≥20 y	-	FFQ	Vitamin C:0.9 (0.6–1.3) for males, 0.6 (0.4–0.97) for females; Vitamin E:0.9 (0.5–1.4) for males, 0.7 (0.4– 1.1) for females	Age (five-year age groups), gender, and race/ethnicity (White, Black, Hispanic, Asian, and other)
Bunin et al. (24)	United States	Case-control (PCC)	155 cases and 155 controls	0–6 y	-	Interviews, FFQ	Vitamin A:0.7 (0.3–1.4) Vitamin C:0.7 (0.4–1.5) Vitamin E:0.7 (0.3–1.3)	Income level.
Delorenze et al. (25)	United States	Case-control (HCC)	748 cases	≥20 y	-	FFQ	Vitamin A:1.07 (0.84–1.57) Vitamin C:0.52 (0.23–1.20) Vitamin E:0.74 (0.55–0.99)	Reporting status, age at diagnosis, treatment, education, marital status, total calories, pack years, and age at first alcoholic drink
Shayanfar et al. (26)	Iran	Case-control (HCC)	128 cases and 256 controls	20–75 y	-	FFQ	Vitamin C:0.482 (0.259–0.899) Vitamin E:0.211 (0.029–1.554)	Age, sex, education, job, smoking, physical activity, and cell phone usage
Wang et al. (27)	China	Case-control (HCC)	343 cases and 343 controls	≥18 y	-	FFQ	Vitamin A:0.10 (0.04–0.30) Vitamin C:0.02 (0.01–0.08) Vitamin E:2.68 (1.03–6.95)	Age, BMI, education, income, smoking, alcohol consumption, family history of cancer, physical activity level, and total energy intake.

BMI, Body mass index; CI, Confidence interval; FFQ, Food frequency questionnaire; HCC, Hospital-based case-control study; PCC, Population-based case-control study; SES, Socio-economic status; Y, Years.

### Vitamin A intake and glioma risk

**Figure 2:**
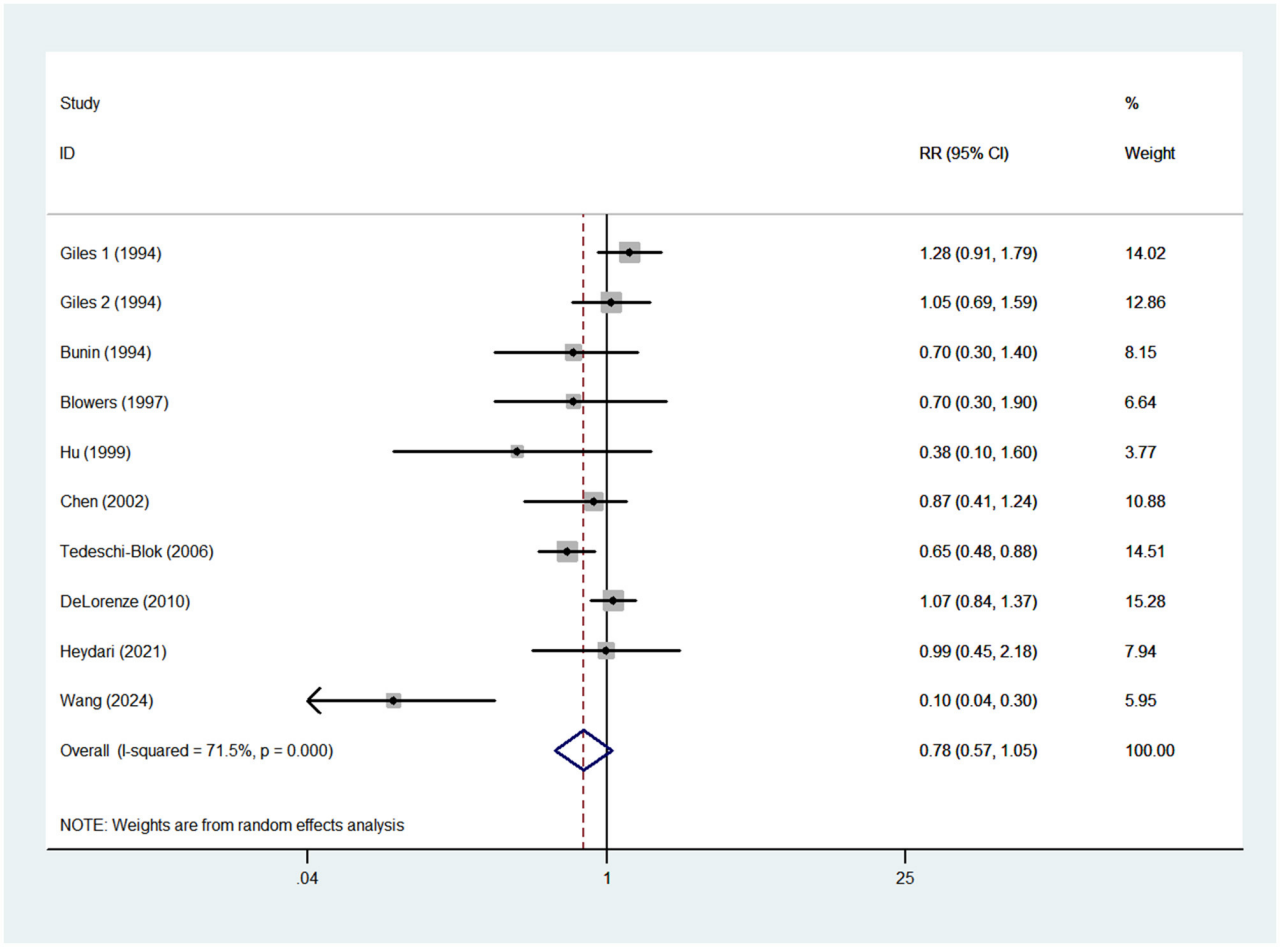
Forest plot for the association between vitamin A intake and risk of glioma.

Nine case-control studies were included in this meta-analysis (13–15, 17–20, 25, 27). As a previous study by Giles et al. (14) reported risk estimates separately for both males and females, 10 risk estimates were entered in the final analysis. Pooled analysis showed that high vitamin A intake was not significantly associated with the risk of glioma (RR = 0.78; 95%CI: 0.57–1.05; *P* = 0.103) (Figure 2), with evidence of significant heterogeneity (I^2^ = 71.5%; *P* < 0.001). Thus, the effect size was assessed using the random-effects model.

### Vitamin C intake and glioma risk

**Figure 3:**
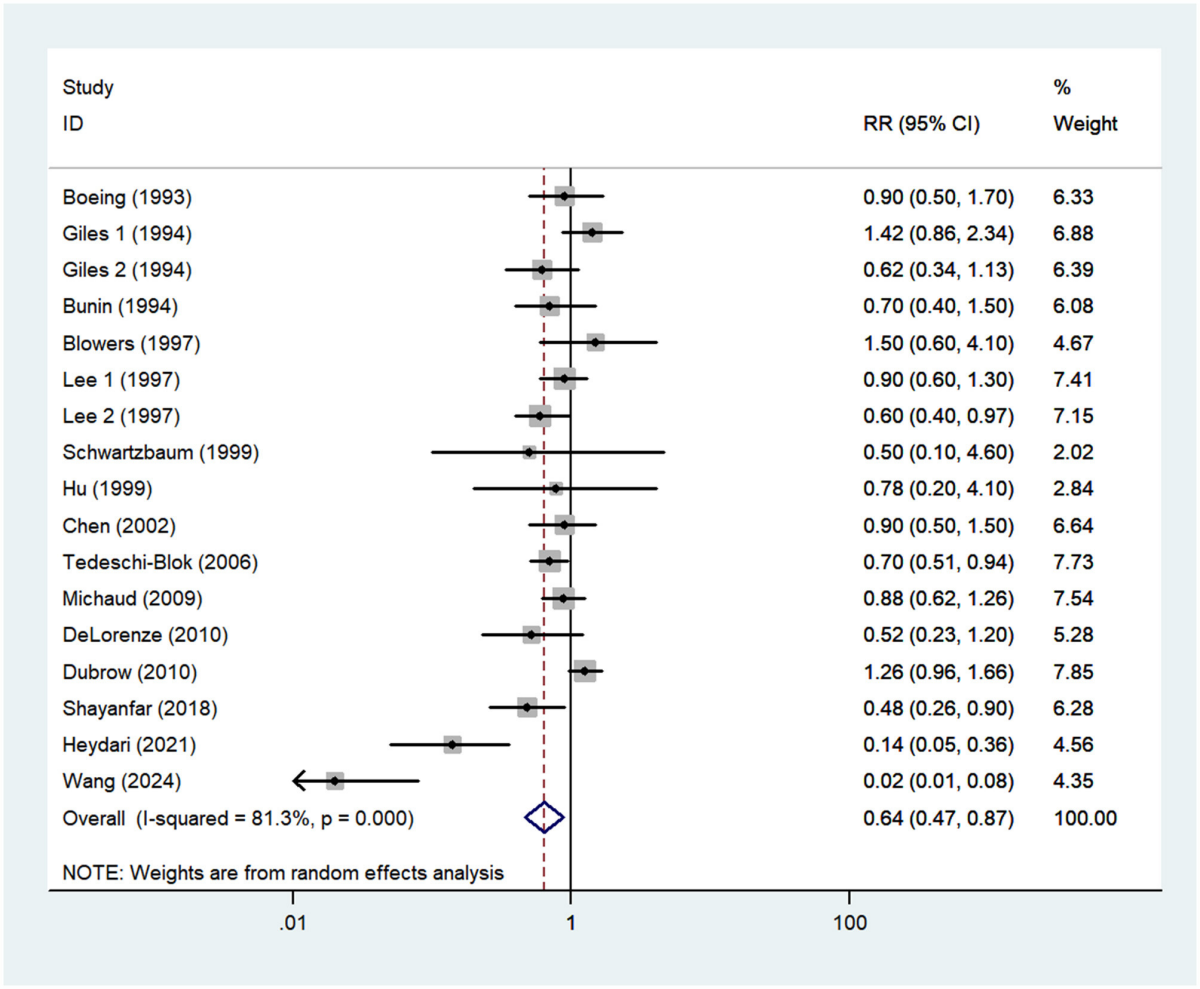
Forest plot for the association between vitamin C intake and risk of glioma.

Fifteen studies, including two prospective cohort (16, 21) and 13 case-control studies (13–15, 17–20, 22–27), were included in this meta-analysis. As the studies by Giles et al. (14) and Lee et al. (23) reported risk estimates separately for both males and females, 17 effect sizes were entered in the final analysis. The pooled RR for the highest vs. lowest category of vitamin C showed a significant inverse association with the risk of glioma (RR = 0.64; 95%CI: 0.47–0.87; *P* = 0.005) (Figure 3). The heterogeneity between included studies was apparent (*P* < 0.001; I^2^ = 81.3%), and thus the random-effects model was used to calculate the combined RR.

### Vitamin E intake and glioma risk

**Figure 4:**
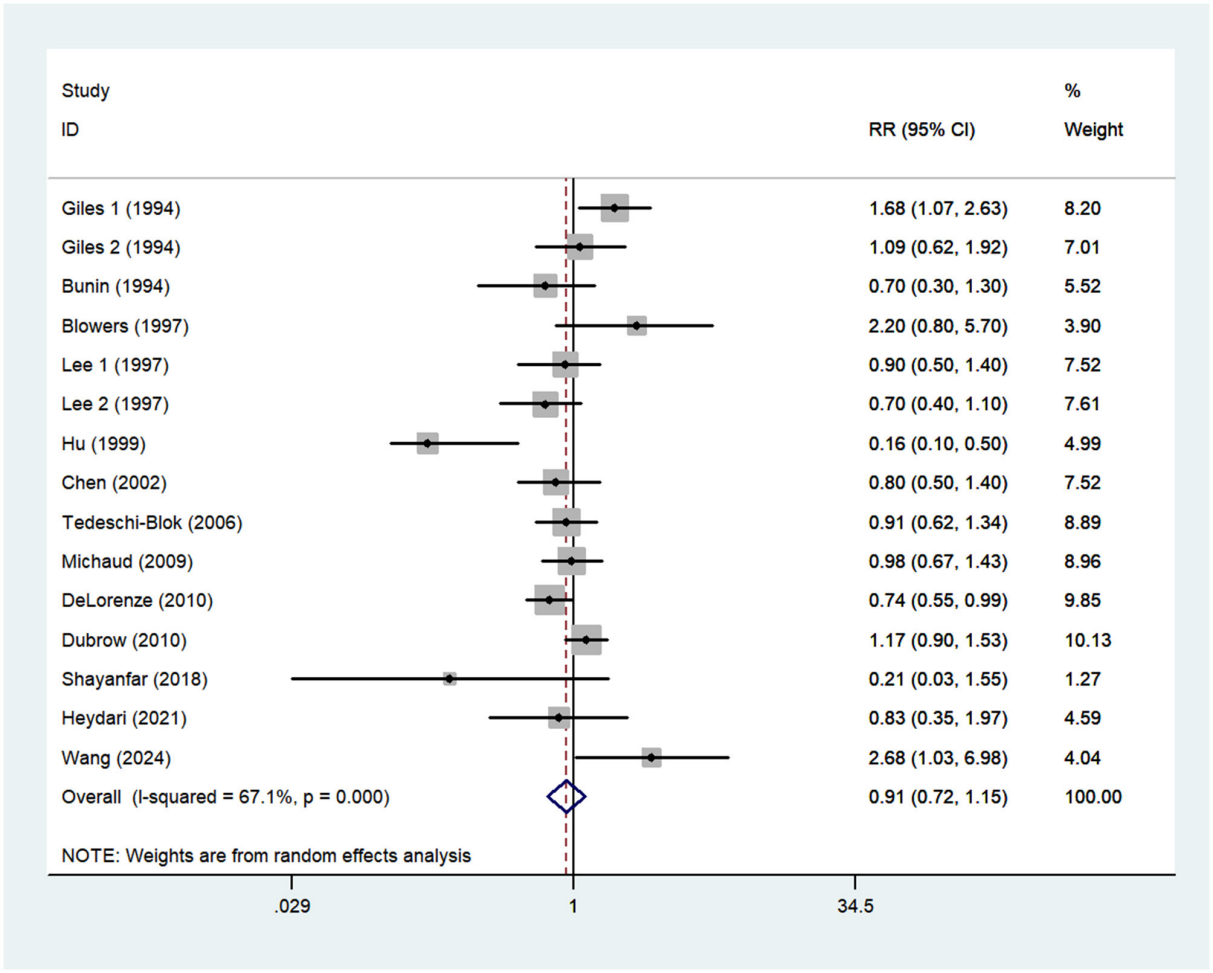
Forest plot for the association between vitamin E intake and risk of glioma.

Thirteen articles (14–21, 23–27) reporting 15 studies were included in the analysis of vitamin E intake and glioma. Figure 4. showed that high intake of vitamin E was not associated with the risk of gioma (RR = 0.91; 95%CI: 0.72–1.15; *P* = 0.429), with significant heterogeneity between studies (I^2^ = 67.1%, *P* < 0.001). Thus, the effect was assessed using the random-effects models.

### Subgroup analyses

Significant between-study heterogeneity was observed between antioxidant vitamins intake and glioma risk. To further explore the probable sources of heterogeneity across studies, we carried out subgroup analyses depending on study design, country, study quality, mean age and sources of control (Table 2). For vitamin A, the results showed that there were no significant association between vitamin A intake and glioma risk in the all subgroups (*P* > 0.05). For vitamin C, we found the protective associations in the population-based case-control studies and study quality score <7. Specifically, for study quality, the results showed that vitamin C intake was statistically significant in the studies with study quality <7 (RR = 0.52, 95%CI: 0.29–0.92, *P* = 0.025), and there was no evidence of heterogeneity (*P* = 0.564; I^2^ = 0.0%). For sources of control, there was an inverse association between vitamin C intake and risk of glioma in the population-based case-control studies (RR = 0.82; 95%CI: 0.68–1.00, *P* = 0.049), with less evidence of heterogeneity (*P* = 0.229; I^2^ = 24.1%). For vitamin E, the results of subgroup analysis showed a significant protective association between vitamin E intake and risk of glioma in the studies with study quality <7 (RR = 0.17; 95%CI:0.08–0.35, *P* < 0.001) and there was no evidence of heterogeneity (*P* = 0.801; I^2^ = 0.0%).

**Table 2:** Subgroup analyses of glioma for the highest vs. lowest category of dietary antioxidant vitamins intake.

**Antioxidant vitamins**	**Subgroup**	**No. of studies**	**RR (95%CI)**	***P*-values**	**Heterogeneity**		
					***P*-values for within groups**	**I^2^ (%)**	***P-*values for between groups**
**Vitamin A**	**Country**						
	Western countries	6	0.93 (0.75–1.16)	0.507	0.081	46.7	0.005
	Other	3	0.34 (0.08–1.51)	0.157	0.002	83.8	
	**Study quality**						
	≥7	8	0.80 (0.59–1.09)	0.155	60.9	73.4	0.22
	<7	1	0.38 (0.09–1.52)	0.171	-	-	
	**Mean age**						
	≥50 y	4	0.87 (0.65–1.16)	0.34	0.095	53	0.724
	<50 y	5	0.62 (0.34–1.13)	0.121	0	80	
	**Sources of control**						
	HCC	4	0.49 (0.18–1.35)	0.167	0	86.2	0.812
	PCC	5	0.89 (0.67–1.16)	0.387	0.081	49	
**Vitamin C**	**Study design**						
	Case-control	13	0.58 (0.40–0.83)	0.003	0	80.3	0
	Cohort	2	1.07 (0.76–1.52)	0.695	0.116	59.4	
	**Country**						
	Western countries	11	0.87 (0.73–1.04)	0.119	0.068	39.9	0
	Other	4	0.18 (0.04–0.83)	0.028	0	90	
	**Study quality**						
	≥7	13	0.65 (0.46–0.90)	0.01	0	83.1	0.127
	<7	2	0.52 (0.29–0.92)	0.025	0.564	0	
	**Mean age**						
	≥50 y	5	0.82 (0.63–1.08)	0.166	0.022	62.1	0.187
	<50 y	9	0.52 (0.29–0.93)	0.027	0	87	
	**Sources of control**						
	HCC	6	0.24 (0.08–0.72)	0.011	0	85.2	0
	PCC	7	0.82 (0.68–1.00)	0.049	0.229	24.1	
**Vitamin E**	**Study design**						
	Case-control	11	0.87 (0.65–1.17)	0.357	0	69.2	0.08
	Cohort	2	1.10 (0.89–1.37)	0.373	0.453	0	
	**Country**						
	Western countries	9	0.97 (0.82–1.16)	0.771	0.078	40.6	0.035
	Other	4	0.56 (0.14–2.20)	0.406	0	85.9	
	**Study quality**						
	≥7	11	1.00 (0.83–1.20)	0.994	0.047	43.4	0
	<7	2	0.17 (0.08–0.35)	0	0.801	0	
	**Mean age**						
	≥50 y	5	0.89 (0.74–1.06)	0.2	0.251	24.4	0.353
	<50 y	8	0.92 (0.57–1.51)	0.752	0	77.2	
	**Sources of control**						
	HCC	5	0.61 (0.27–1.42)	0.257	0	81.9	0.016
	PCC	6	0.99 (0.78–1.27)	0.955	0.114	39.7	

HCC, Hospital-based case-control study; PCC, Population-based case-control study.

### Quality assessment

The quality of included studies using Newcastle-Ottawa criteria is shown in Table 3. When included studies received a score of seven or higher, they would be deemed to be of relatively higher quality (13, 14, 16–25, 27).

**Table 3:** Dietary antioxidant vitamins intake and glioma: assessment of study quality.

**References**	**Selection**				**Comparability**		**Outcome**			
	**1**	**2**	**3**	**4**	**5A**	**5B**	**6**	**7**	**8**	**Score**
Boeing et al. (13)	^*^	^*^		^*^	^*^	^*^	^*^	^*^		7
Giles et al. (14)	^*^	^*^		^*^	^*^		^*^	^*^		7
Hu et al. (15)	^*^	^*^	^*^		^*^		^*^	^*^		6
Dubrow et al. (16)	^*^	^*^		^*^	^*^	^*^	^*^	^*^		7
Tedeschi-Blok et al. (17)	^*^	^*^		^*^	^*^	^*^	^*^	^*^	^*^	8
Heydari et al. (18)	^*^	^*^		^*^	^*^		^*^	^*^	^*^	7
Blowers et al. (19)	^*^	^*^		^*^	^*^		^*^	^*^	^*^	7
Chen et al. (20)	^*^	^*^		^*^	^*^		^*^	^*^	^*^	7
Michaud et al. (21)	^*^	^*^		^*^	^*^	^*^	^*^	^*^	^*^	8
Schwartzbaum et al. (22)	^*^	^*^	^*^	^*^	^*^		^*^	^*^	^*^	8
Lee et al. (23)	^*^	^*^		^*^	^*^		^*^	^*^	^*^	8
Bunin et al. (24)	^*^	^*^		^*^	^*^	^*^	^*^	^*^	^*^	8
Delorenze et al. (25)	^*^	^*^	^*^	^*^	^*^		^*^	^*^		7
Shayanfar et al. (26)	^*^	^*^		^*^	^*^		^*^	^*^		6
Wang et al. (27)	^*^	^*^	^*^	^*^	^*^		^*^	^*^		7

^*^For case-control studies, 1 indicates cases independently validated; 2, cases are representative of population; 3, community controls; 4, controls have no history of brain cancer; 5A, study controls for age; 5B, study controls for additional.

factor(s); 6, ascertainment of exposure by blinded interview or record; 7, same method of ascertainment used for cases and controls; and 8, non-response rate the same for cases and controls. For cohort studies, 1 indicates exposed cohort.

truly representative; 2, non-exposed cohort drawn from the same community; 3, ascertainment of exposure; 4, outcome of interest not present at start; 5A, cohorts comparable on basis of age; 5B, cohorts comparable on other factor(s);

6, quality of outcome assessment; 7, follow-up long enough for outcomes to occur; and 8, complete accounting for cohorts.

### Publication bias

The visual inspection of funnel plots revealed little evidence of asymmetry (Supplementary Figures 1–3). Similarly, Egger's and Begg's tests for publication bias were not statistically significant (highest compared with lowest intake: vitamin A, Egger's test: *P* = 0.112; Begg's test: *P* = 0.074; vitamin E, Egger's test: *P* = 0.537; Begg's test: *P* = 0.729). However, in this study, Egger's test showed statistical evidence of bias for vitamin C and glioma (*P* = 0.043), but Begg's test did not (*P* = 0.064). Therefore, we used the trim and fill analysis to re-estimate the combined risk estimates (Supplementary Figure 4). After performing trim and fill analysis, five studies were added to the funnel plot, which showed a low degree of asymmetry and no significant change in the pooled risk estimates (RR = 0.47; 95%CI: 0.32–0.67, *P* < 0.001).

### Sensitivity analysis

The result of sensitivity analysis showed that no particular study had the significant impact on the associations between dietary antioxidant vitamins intake and glioma risk when we excluded individual study at a time, indicating robust and reliable results (Supplementary Figures 5–7).

## Discussion

The existing literature regarding the links between dietary antioxidant vitamins intake and risk of glioma are scarce and controversial. To our knowledge, this is the latest systematic review and meta-analysis synthesizing the existing evidence on the effect of high antioxidant vitamins intake on glioma. In the present study, pooled results showed that high intake of vitamin C was significantly associated with a lower risk of glioma, whereas high intake of vitamin A and vitamin E were not associated with the risk of glioma. Sensitivity analysis showed that no particular study significantly affected the summary effects. Findings from this study have important implications for the prevention of glioma, as it add strong and consistent evidence to support the favorable influence of high vitamin C intake in reducing glioma risk. Also, our study also provide further reinforcement to the previous meta-analysis (28).

Although the incidence of glioma is extremely low, its high mortality rate and poor prognosis have gained increasing concern in recent years. Thus, it is critical to identify the effect of modifiable risk factors, such as diet on glioma. To date, the considerable attentions have been paid to the effect of dietary antioxidant vitamins on glioma risk in humans (13–27). However, findings from the aforementioned studies remain controversial. A previous meta-analysis published in 2015 by Lv et al., high intake of dietary vitamin A was significantly associated with reduced risk of glioma (RR = 0.80, 95% CI = 0.6–0.98, *P* = 0.014) (36). In contrast to previous meta-analysis, our findings showed a null association between dietary vitamin A intake and risk of glioma (RR = 0.78; 95%CI: 0.57–1.05; *P* = 0.103).The reasons for this discrepancy are hard to fully elucidate. But, there are several scenarios to explain this null association. First, all included studies were case-control design. Thus, selection bias was inevitably introduced. Second, dietary vitamin A intake was assessed by FFQs, for which there is an inherent recall bias. Third, this discrepancy might be explained by the significant heterogeneity (I^2^ = 71.5%) of studies in this meta-analysis. Moreover, we included more studies than the previous meta-analysis. In short, above-mentioned these could explain, at least in part, these discrepant results. More prospective cohort studies are required to ascertain the association between dietary antioxidant vitamins and glioma risk.

Recently, the effect of vitamin C intake on glioma has received widespread attention from researchers (37). So far, however, epidemiological evidence concerning the association between vitamin C intake and glioma risk has been inconsistent (13–17, 23), with the majority of published studies, showing that higher consumption of vitamin C was not significantly associated with the risk of glioma (13, 14). However, an earlier meta-analysis of 13 articles illustrated that higher intake of vitamin C was significantly associated with a reduced risk of glioma, especially among the Americans (28). In coherence with the aforementioned findings, our results further confirmed the strong protective association between high vitamin C intake and glioma risk when the results of all included studies were pooled. However, data from three US prospective cohort studies found essentially null association between vitamin C intake and glioma risk (16). Still, as one of the most important component of fruits and vegetables, vitamin C has exerted its anti-carcinogenic properties. In fact, there are several potential explanations for the beneficial effect of high vitamin C intake on glioma. First, the protective effect of vitamin C intake on glioma may be attributed in part to its antioxidant effect. Compared with other tissues, brain tissues are one of the most oxidative environments and thus susceptible to radiation damage, resulting in DNA loss and tumor development (38, 39). Previous research has shown that vitamin C, as one of the most common antioxidants, can reduce oxidative DNA damage and protect normal brain tissues from radiation damage, thereby leading to better survival (39). In addition, it is well established that vitamin C is a water soluble scavenger of hydroxyl radicals, which can inhibit oxidative DNA lesions such as 8-hydroxydeoxyguanosine (40). Second, higher dietary intake of antioxidants, as for instance vitamin C can act as reducing agents to prevent oxidative reactions by either scavenging reactive oxygen species (ROS) or inhibiting cellular signaling enzymes such as protein kinase C (PKC) (41). Uht et al. and Martín et al., found that proliferation of malignant gliomas could be induced by PKC isoforms through several signaling pathways (42, 43). Third, experiments have shown that vitamin C may inhibit the formation of N-nitroso compounds (NOCs) from nitriates in humans (44). NOCs have been found in brain tissue and are involved in the pathogenesis of brain tumors (45). Koestner et al. also confirmed that NOCs could induce glioma by reducing the repair efficiency after DNA damage (46). Fourth, Hung et al.'s study found that vitamin C could inhibit and reduce N-acetyltransferase activity and the formation of 2- aminofluorence-DNA adduct in rat C-6 glioma cells in a dose-dependent manner (47). Finally, cell experiments have already shown that vitamin C can inhibit the growth of glioblastoma through the caspase-3 death pathway and then assist the treatment of glioblastoma with methotrexate (48). Collectively, our findings provide vigorous evidence to support the conclusion that high intake of vitamin C was associated with a reduced risk of glioma.

In our analyses, non-significant inverse association was found between dietary vitamin E intake and risk of glioma. Our findings are in line with a previous meta-analysis of vitamin E intake and risk of glioma, indicating that vitamin E intake is not associated with the risk of glioma (49). Nevertheless, a recent hospital-based case-control study by Wang et al., concluded that higher intake of dietary vitamin E may be associated with an increased risk of glioma (27). But, in dose-response analyses, Wang and his colleagues found that this association was not statistically significant. Concurrently, in an earlier case-control study, Giles et al., also found a positive association between vitamin E intake and glioma in Australian men (OR = 1.68,95%CI:1.07–2.63) (14). Of particularly concern is that laboratory studies have shown that dietary antioxidants, including vitamin E intake, can enhance the growth restriction of glioma cells (50, 51). Thus, several hypotheses could be put forward to explain the null association. First, the majority of included studies were case-control design. As is known to all, case-control studies may be subject to recall bias and selection bias, leading to an underestimation or overestimation of the association between vitamin E intake and glioma risk. Second, no significant association may also be associated with different clinical subtypes of glioma. In Chinese population, Wang et al. found that dietary vitamin E had different effects on gliomas of different pathological subtypes and pathological grades (27). However, given the limited data in the included studies, we were unable to analyze the effect of vitamin E intake on clinical subtypes of glioma. Overall, as already discussion above, these might explain the observed non-significant association between vitamin E intake and risk of glioma.

While our results showed a negative association between high vitamin C intake and glioma risk, this meta-analysis also found the significant between-study heterogeneity (I^2^ = 81.3%; *P*_forheterogeneity_ <0.001). Subgroup analyses stratified by study design, country, study quality, mean age and sources of control were performed to examine the sources of heterogeneity. The results of subgroup analyses showed that significant heterogeneity could be partly attributable to the differences in study design and sources of control. The results of case-control studies indicated that vitamin C intake could significantly reduce the risk of glioma (RR = 0.58; 95%CI: 0.40–0.83, *P* = 0.003). In contrast, we found that vitamin C intake had no significant effect on glioma in cohort studies (RR = 1.07; 95%CI: 0.76–1.52, *P* = 0.695). On the one hand, only two prospective cohort studies were included in the present study, and the results were insignificant. Thus, this may limit the significance of the combined results to a certain extent. On the other hand, the vast majority of published studies on the association between vitamin C and glioma have included a relatively small number of cases, which limited the statistical power to detect the exact association. We also speculated that sources of control might explain the heterogeneity observed in our study. In the subgroup of sources of control, there was an inverse association between vitamin C intake and risk of glioma in the population-based case-control studies (RR = 0.82; 95%CI: 0.68–1.00, *P* = 0.049), with less evidence of heterogeneity (*P* = 0.229; I^2^ = 24.1%). The overall heterogeneity has decreased from 81.3% to 24.1%. There are several possible explanations for this heterogeneity. First, the half of included studies were hospital-based case-control studies. As such, selection bias might contribute the significant heterogeneity (52). Second, compared with other included studies, Hu et al.'s study only included the consumption of 57 food items and might not be able to obtain more accurate dietary vitamin C intake, resulting in considerable heterogeneity. Third, although the ORs were from the highest category (taking the lowest category as the reference), different case-control studies divided vitamin C intake into different intervals. This could also lead to the significant heterogeneity. Finally, there was still some degree of heterogeneity in subgroup analyses, suggesting that there may be other unknown or unidentified confounding factors.

### Strengths and limitations

There are several advantages to this study. First, to our knowledge, this study is the latest systematic review and meta-analysis exploring the associations between dietary antioxidant vitamins intake and the risk of glioma. We not only have an confirmation and timely update of the earlier meta-analyses (27, 36, 49), but also further add to the accumulating evidence on the favorable effect of vitamin C intake against glioma. Also of note, our study included more glioma cases and participants than the previous meta-analyses, allowing for more credible results. Second, the rigorous literature search, study selection and data extraction were conducted by two independent reviewers, thereby increasing the validity of our results. Furthermore, we also rejected several articles that had been included in the previous meta-analyses but involved other brain tumors. Third, the cases of glioma have been diagnosed through clinical manifestations and pathological section, avoiding misdiagnosis. Fourth, no signs of publication bias were evident in the funnel plot, and the statistical test (e.g., Egger's and Begg's tests) for publication bias was non-significant. Fifth, we discussed in depth the sources of heterogeneity and improved the accuracy of the significant results. Nevertheless, some potential limitations should also be discussed when interpreting the results of this study. First of all, owing to the observational nature of all included studies, confounding factors are often of concern. Thus, we cannot entirely rule out the probability that these findings were susceptible to recall and selection biases. Also, antioxidant vitamins intake was evaluated through questionnaire and measurement errors in the present meta-analysis were inevitable. Collectively, the imprecise measurement of antioxidant vitamins intake might have attenuated the true associations. Further studies, particularly well-designed prospective studies, were therefore needed to confirm these findings. Second, the adjustment variables in all included studies were inconsistent, leading to large differences in the values of OR and RR. As a result, the pooled risk estimates data may suffer from differing degrees of completeness and accuracy. Third, given relatively rare incidence of glioma and insufficient data, we were unable further to explore the associations between dietary antioxidant vitamins intake and glioma subtypes, including glioblastoma and oligodendro-glioma. Fourth, although high vitamin C intake was associated with a lower risk of glioma, the results should be interpreted with caution due to the significant heterogeneity observed. Because we couldn't ascertain and explain the sources of inter-study heterogeneity sufficiently, after performing subgroup analyses. Fifth, we could not conduct a dose-response analysis for dietary antioxidant vitamins with glioma risk due to the limited data reported in each individual study. Finally, considering that the existing studies were primarily carried out in the United States or Western countries, the extrapolation of the findings might be limited.

## Conclusion

To conclude, this study showed that high vitamin C intake was associated with a reduced risk of glioma. In addition, there were no statistically significant associations between vitamin A, vitamin E and glioma risk. Our findings provide compelling evidence of a significant inverse association between vitamin C intake and risk of glioma, and emphasize the role of antioxidant vitamins intake for the prevention of glioma. However, due to the insufficient number of studies and lack of the dose-response relationship, our findings should be interpreted with caution. Further studies, particularly prospective cohort studies, are urgently required to validate and strengthen these findings and provide a more comprehensive understanding of the associations between dietary antioxidant vitamins and glioma risk.

## Data availability statement

The original contributions presented in the study are included in the article/Supplementary material, further inquiries can be directed to the corresponding author.

## Author contributions

Y-JN: Conceptualization, Writing – original draft. Y-QH: Data curation, Investigation, Writing – review & editing. LY: Data curation, Investigation, Writing – review & editing. X-YZ: Data curation, Methodology, Writing – review & editing. QZ: Data curation, Formal analysis, Funding acquisition, Writing – review & editing. LS: Formal analysis, Funding acquisition, Methodology, Writing – review & editing. LZ: Conceptualization, Supervision, Writing – original draft, Validation, Writing – review & editing.
