# Supplementary material for: Associations between dietary antioxidant vitamins and risk of glioma: an updated systematic review and meta-analysis of observational studies
Source: Front Nutr. 2024 Aug 6;11:1428528. doi: 10.3389/fnut.2024.1428528 (PMC11333925; doi:10.3389/fnut.2024.1428528)
Supplement: Supplementary file 1 [file Table_1.DOC]

**Search terms in PubMed, Web of Science, Emabase and Scopus**
((“diet”[all fields] OR “nutrition” [all fields] OR “nutrient”[all fields] OR “vitamin C”[all fields] OR “vitamin E”[all fields] OR “vitamin A”[all fields] OR “antioxidants”[all fields]) AND (“glioma”[all fields] OR “glioblastoma” [all fields] OR “brain cancer”[all fields] OR “brain tumor”[all fields]))

**Search terms in CNKI and Wanfang Data**

(饮食 OR 营养OR 营养素 OR 氧化性维生素 OR 维生素A OR 维生素C OR 维生素E) and (脑癌 OR 脑肿瘤 OR 神经胶质瘤）

**Search terms in other sources**

Manual searches in the reference lists from the selected articles and reviews or meta-analyses were performed to identify the potentially eligible studies
