# Supplementary material for: Associations between dietary antioxidant vitamins and risk of glioma: an updated systematic review and meta-analysis of observational studies
Source: Front Nutr. 2024 Aug 6;11:1428528. doi: 10.3389/fnut.2024.1428528 (PMC11333925; doi:10.3389/fnut.2024.1428528)
Supplement: Supplementary file 2 [file Table_2.DOC]

Table S2. PICOS criteria for inclusion and exclusion of studies

| **Population** | **Adults** |
| --- | --- |
| Exposure | Antioxidant vitamins intake |
| Comparison | Highest vs. lowest categories of exposure |
| Outcomes | Glioma |
| Study design | Cohort, case-control or nested case-control studies |

PICOS, participant, intervention(exposure), comparison, outcome, and study design
