# Supplementary figures and images for: Associations between dietary antioxidant vitamins and risk of glioma: an updated systematic review and meta-analysis of observational studies
Source: Front Nutr. 2024 Aug 6;11:1428528. doi: 10.3389/fnut.2024.1428528 (PMC11333925; doi:10.3389/fnut.2024.1428528)

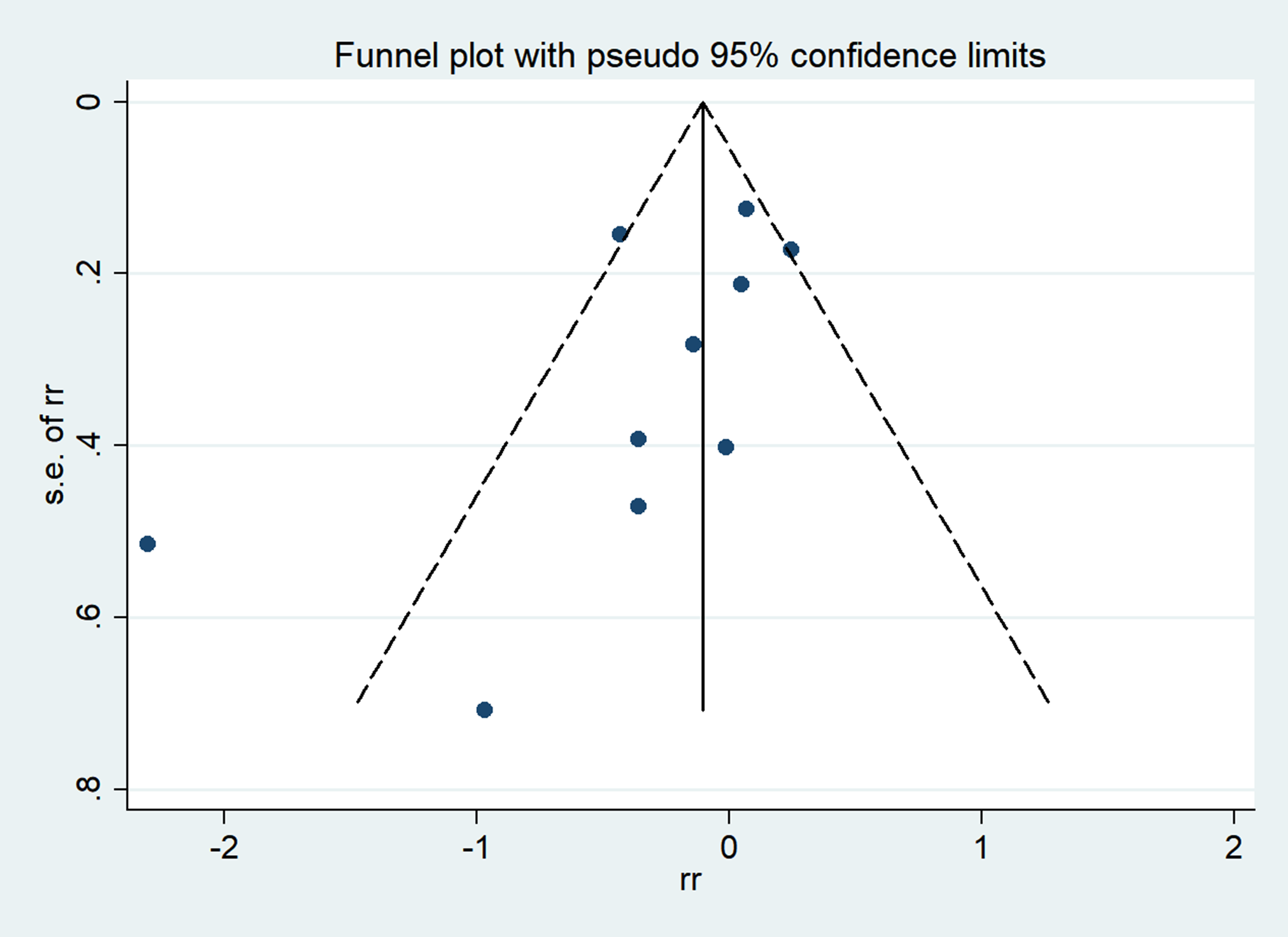

Supplement: Supplementary file 3 [file Image_1.TIF]

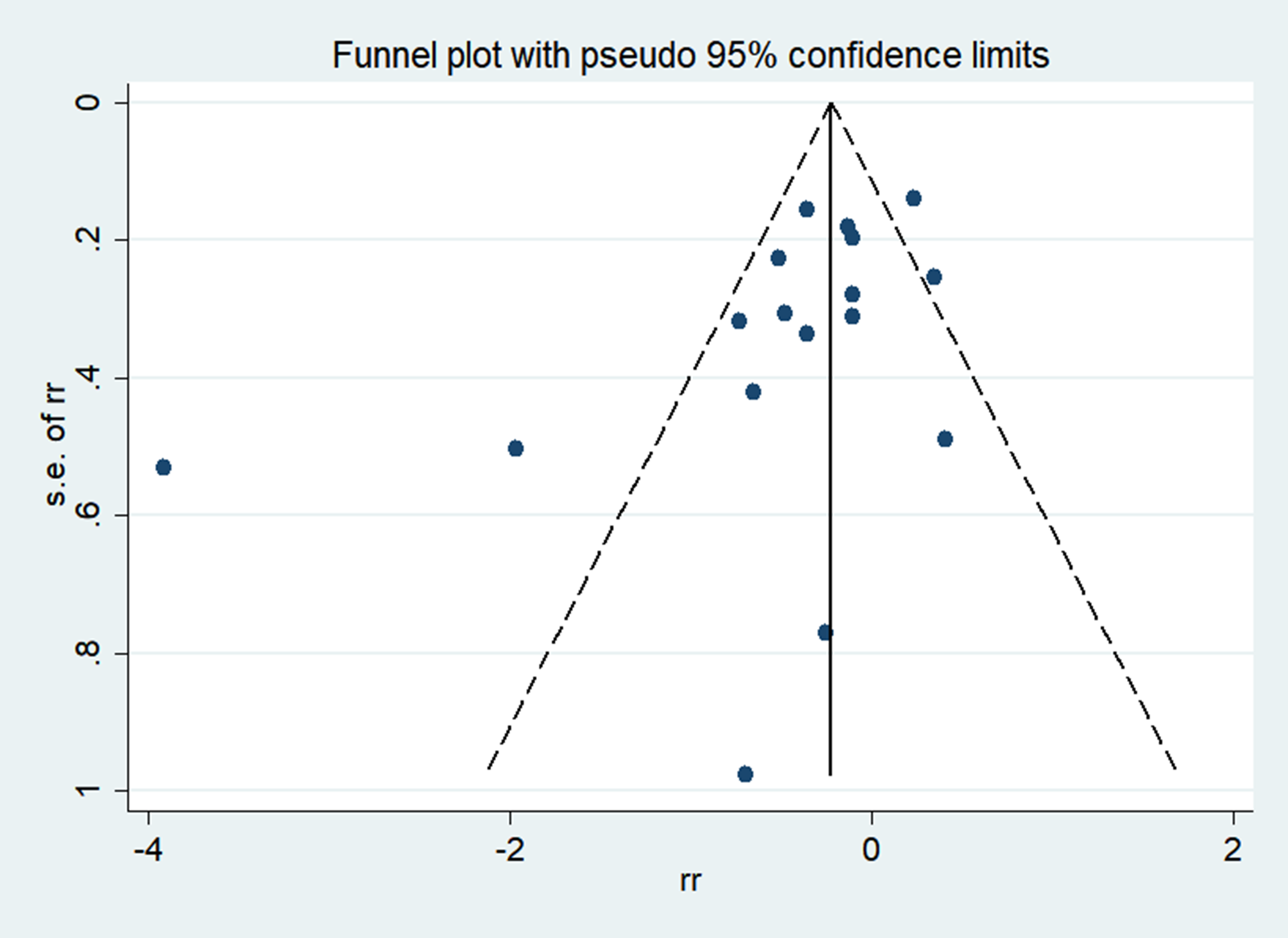

Supplement: Supplementary file 4 [file Image_2.TIF]

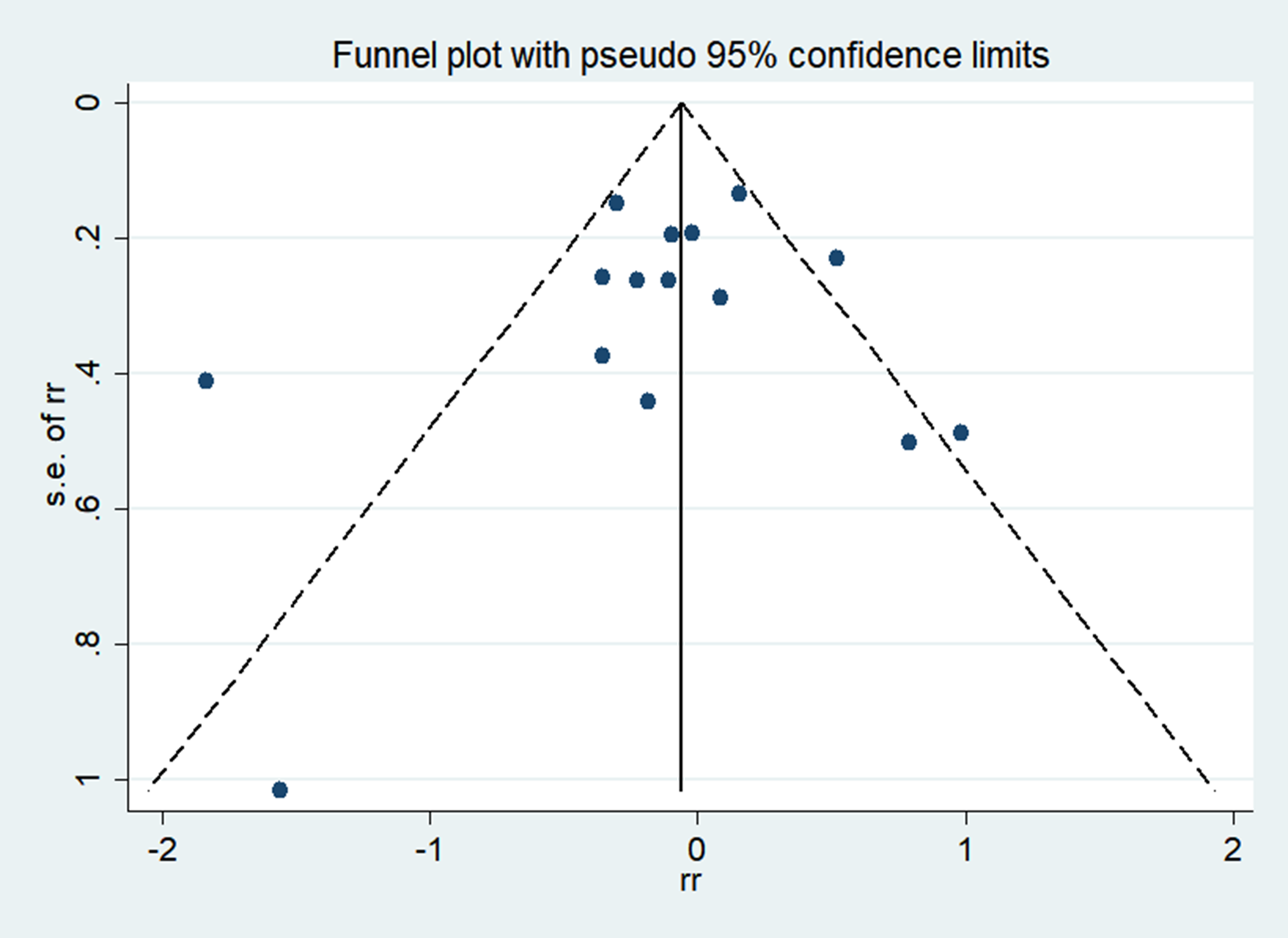

Supplement: Supplementary file 5 [file Image_3.TIF]

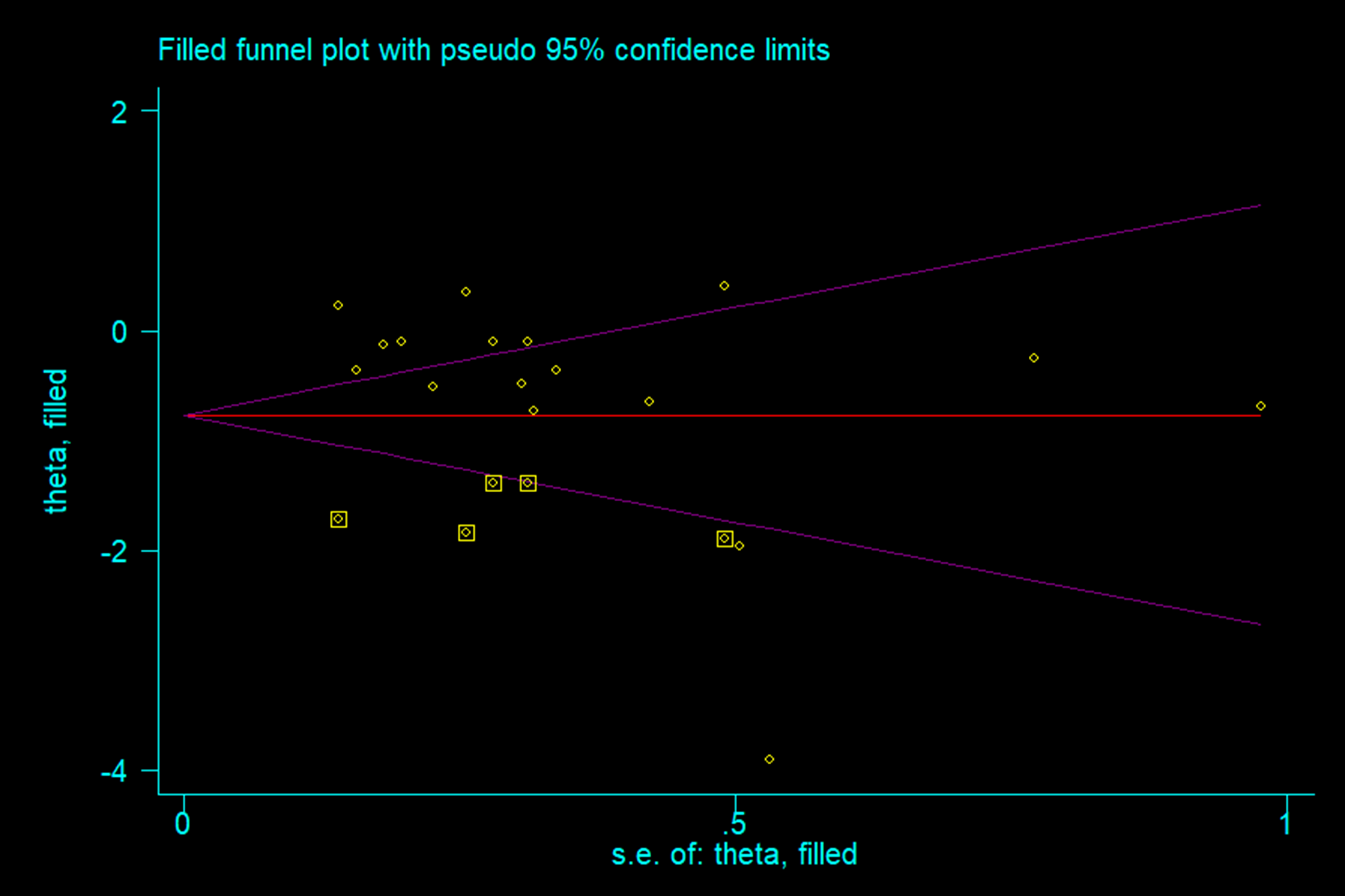

Supplement: Supplementary file 6 [file Image_4.TIF]

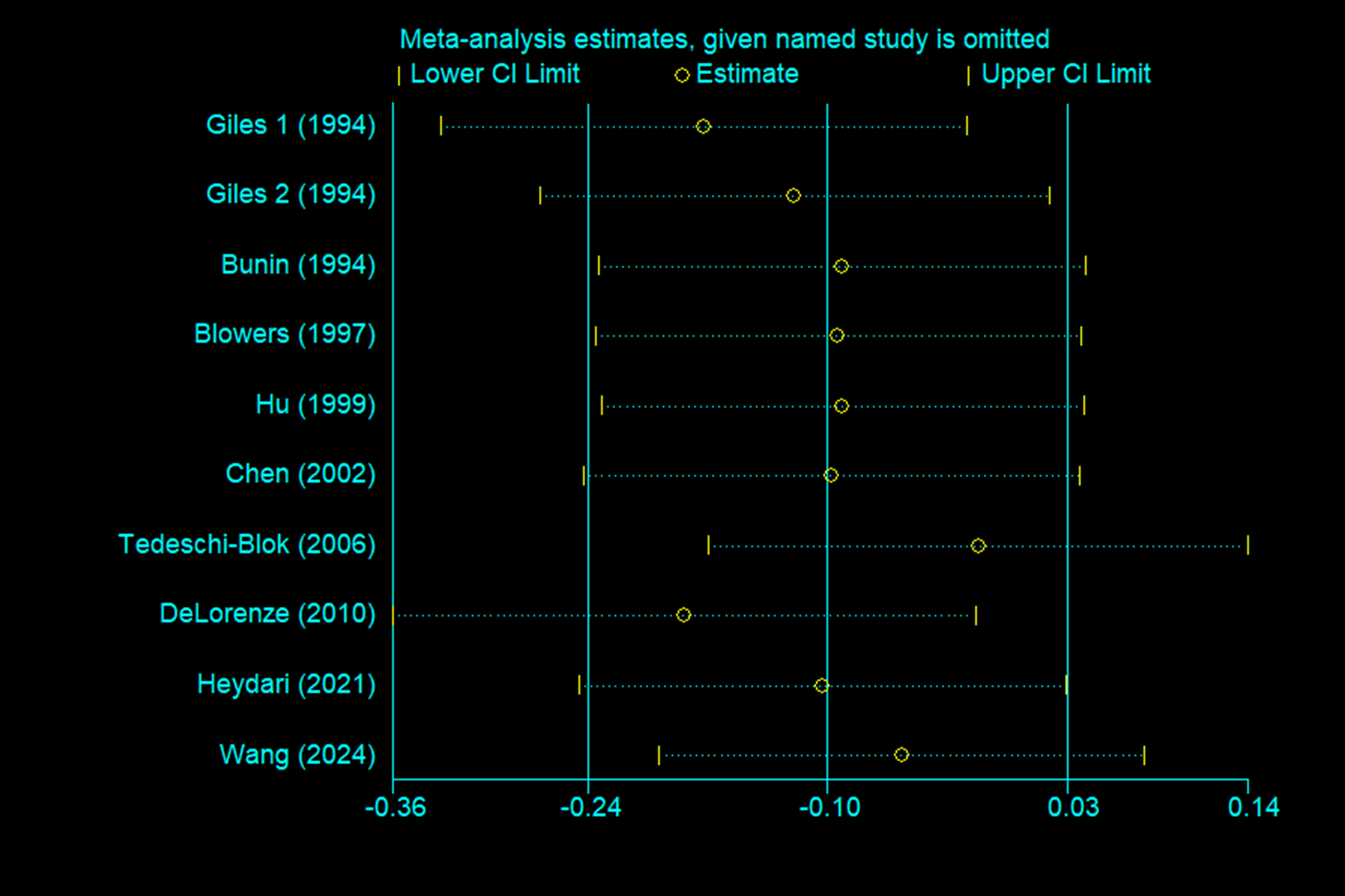

Supplement: Supplementary file 7 [file Image_5.TIF]

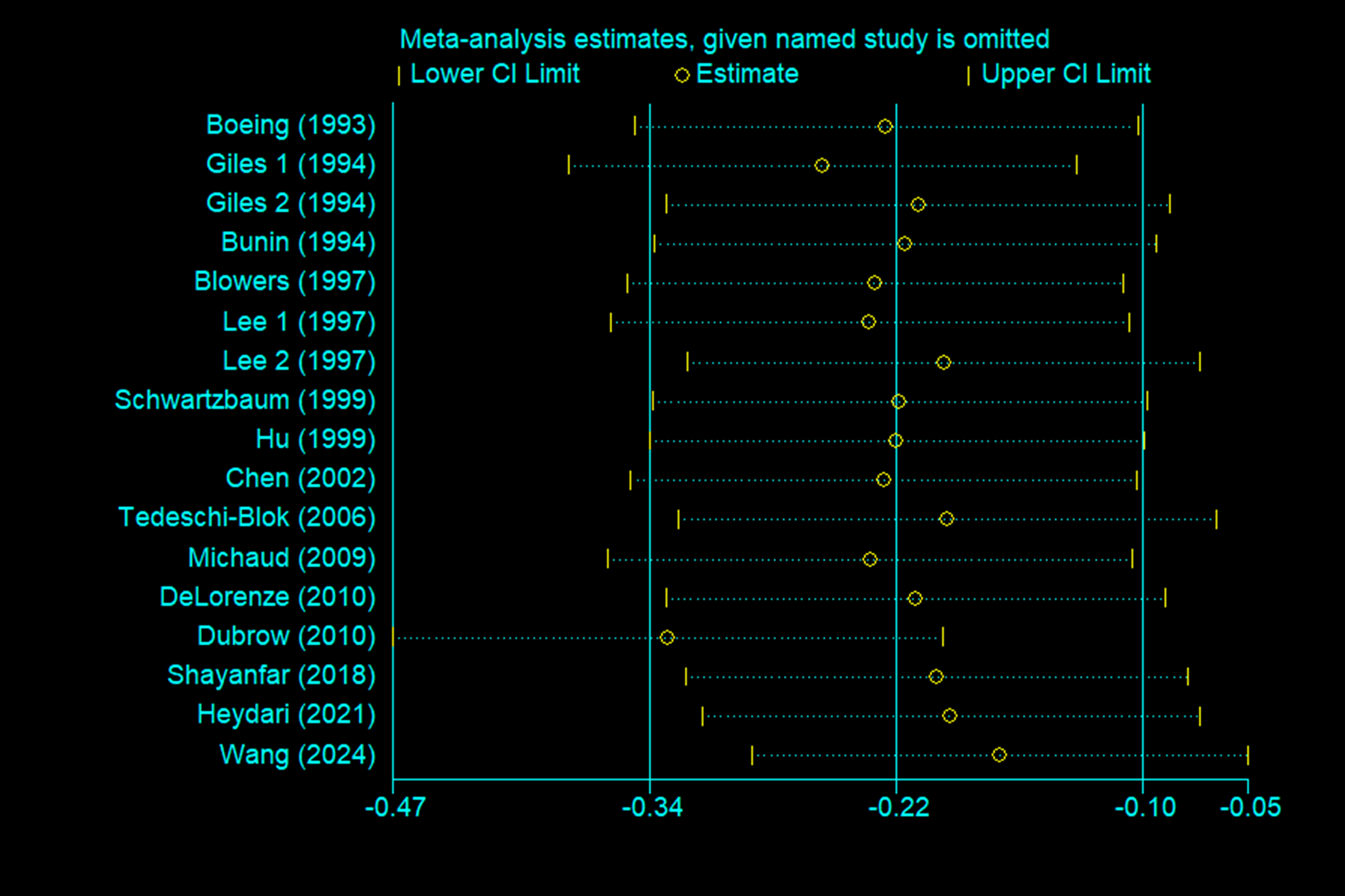

Supplement: Supplementary file 8 [file Image_6.TIF]

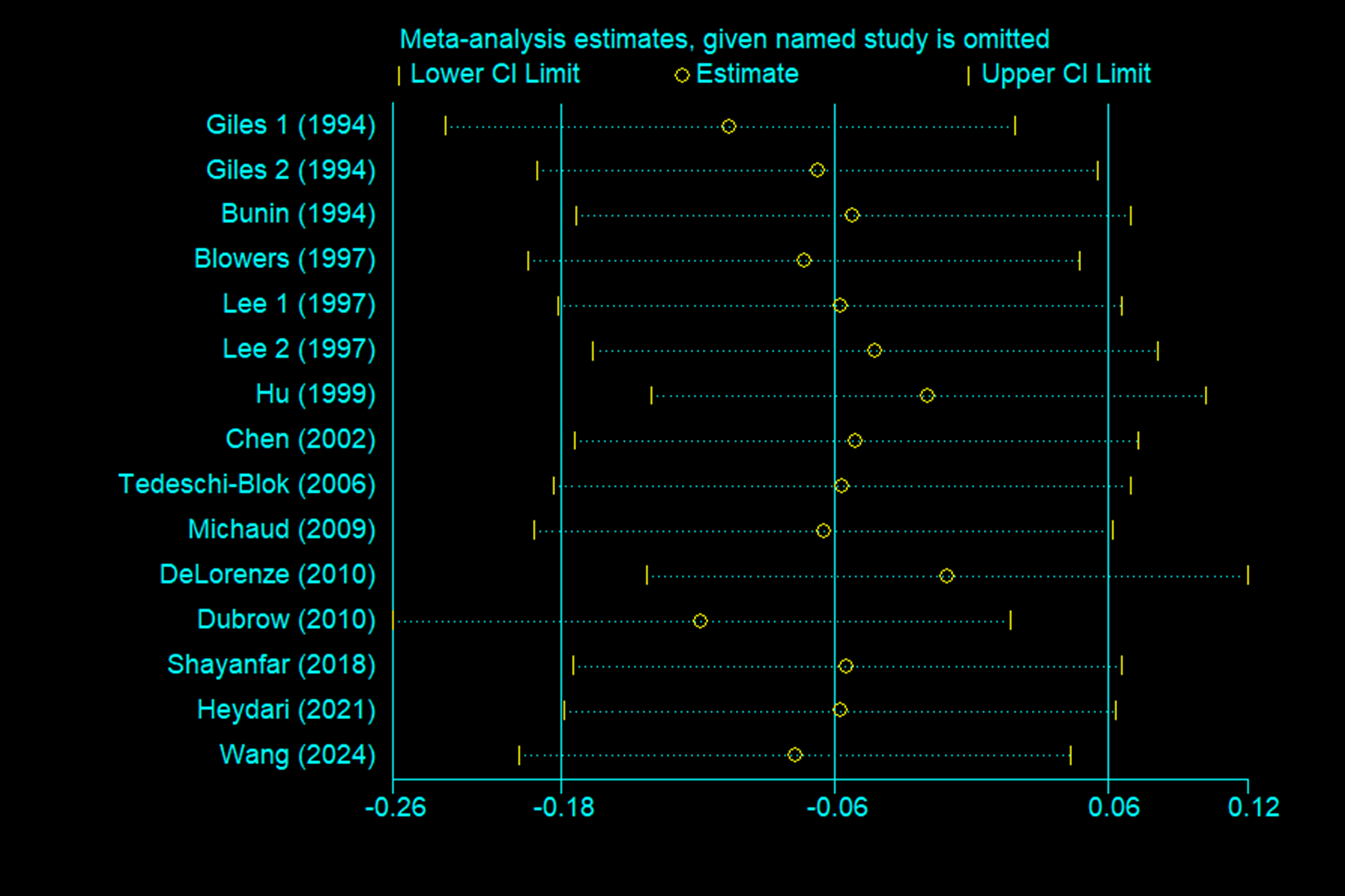

Supplement: Supplementary file 9 [file Image_7.TIF]
